# Detection and Mitigation of IoT-Based Attacks Using SNMP and Moving Target Defense Techniques

**DOI:** 10.3390/s23031708

**Published:** 2023-02-03

**Authors:** Rajakumaran Gayathri, Shola Usharani, Miroslav Mahdal, Rajasekharan Vezhavendhan, Rajiv Vincent, Murugesan Rajesh, Muniyandy Elangovan

**Affiliations:** 1 Vellore Institute of Technology, Chennai Campus, Chennai 600127, India; 2 Department of Control Systems and Instrumentation, Faculty of Mechanical Engineering, VSB-Technical University of Ostrava, 17, Listopadu 2172/15, 70800 Ostrava, Czech Republic; 3 Vellore Institute of Technology, Vellore 632014, India; 4 Department of R&D, Bond Marine Consultancy, London EC1V 2NX, UK

**Keywords:** cloud computing, IoT, cloud security, access control list, simple network monitoring protocol, Amazon Web Service (AWS), moving target defense (MTD)

## Abstract

This paper proposes a solution for ensuring the security of IoT devices in the cloud environment by protecting against distributed denial-of-service (DDoS) and false data injection attacks. The proposed solution is based on the integration of simple network management protocol (SNMP), Kullback–Leibler distance (KLD), access control rules (ACL), and moving target defense (MTD) techniques. The SNMP and KLD techniques are used to detect DDoS and false data sharing attacks, while the ACL and MTD techniques are applied to mitigate these attacks by hardening the target and reducing the attack surface. The effectiveness of the proposed framework is validated through experimental simulations on the Amazon Web Service (AWS) platform, which shows a significant reduction in attack probabilities and delays. The integration of IoT and cloud technologies is a powerful combination that can deliver customized and critical solutions to major business vendors. However, ensuring the confidentiality and security of data among IoT devices, storage, and access to the cloud is crucial to maintaining trust among internet users. This paper demonstrates the importance of implementing robust security measures to protect IoT devices in the cloud environment and highlights the potential of the proposed solution in protecting against DDoS and false data injection attacks.

## 1. Introduction

Cloud computing plays an important and interconnected role amongst today’s trending technologies related to the Internet of Things (IoT), machine learning (ML), deep learning (DL), and cyber-physical systems (CPS). All these major domains require cloud platforms as mandatory ones for the storage and processing of data from sensors and devices and to provide possible suggestions. All the massive applications related to smart cities, surveillance, or smart transports also depend on cloud platforms either for storing or retrieval of their data as it is more flexible and convenient to access the information anywhere around the globe instead of maintaining it in a local environment [1,2]. The interrelated preferences of such critical applications push the cloud to ensure the maximum level of security [3]. The target of this paper is to identify the wide category of security violations targeting IoT data in rest and motion. The security framework is formulated for the identified attack categories by adopting effective methodologies and algorithms.

The year 2020 accelerated the prevalence of digital technology and the cloud due to the benefits of connecting all the needs in spite of several ongoing challenges. As connectivity seems to be increasingly necessary for individuals pursuing work from home during the COVID-19 pandemic, digital transformation and data transmitted by IoT devices have begun their rapid blooming phase. The data transmitted by IoT devices paved a critical role in order to optimize business processes, analyzing patterns, streamlining processes, and understanding everyday business routines [4,5]. The digital transformation routed us to today’s integrated platform of cloud and IoT. The combined solutions of cloud and IoT help in supporting data capture with real-time control and intelligence monitoring.

The applications of cloud-assisted IoT technologies range widely from:

- Smart environments and smart cities;
- Telemedicine;
- Intelligent transportation system;
- Smart Parking;
- Drones co-ordination;
- Healthcare.

**Figure 1:**
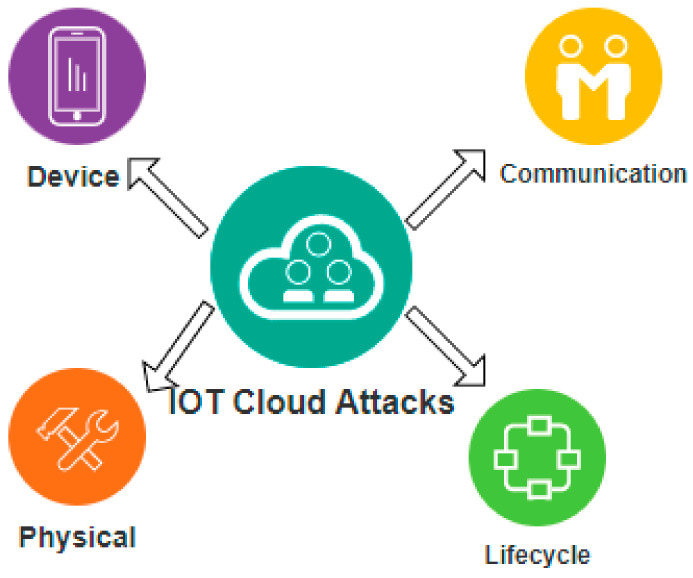
IoT Attack Categories.

**Figure 2:**
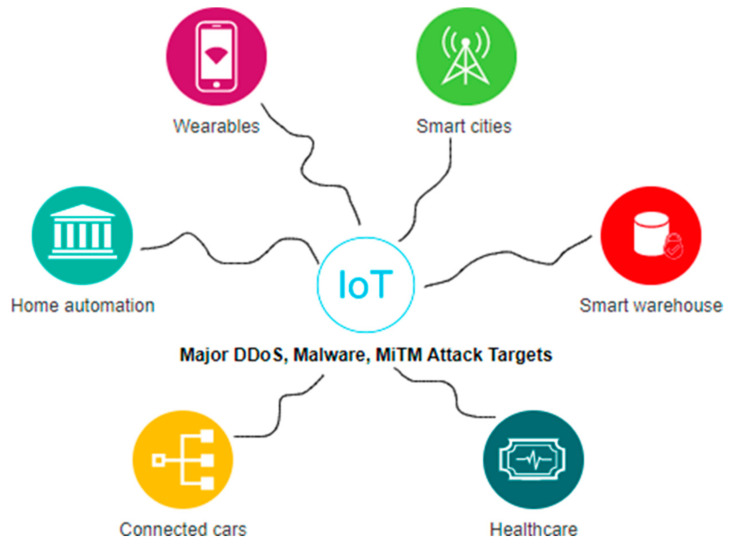
Major IoT Attack Targets.

The growing nature and interconnectivity of the internet make the IoT platform more scalable and easily adaptable. IoT has put its marks in the fields of food production, the manufacturing industry, the finance sector, and the healthcare domain. Day by day, more automation techniques are getting merged into IoT platforms to enhance a high degree of comfort and convenience for internet users. More and more organizations are migrating towards IoT, which in turn, increases the count of IoT devices. Collection and monitoring such a volume of IoT devices prove challenging as it suffers from severe security violations. Hence the attack surfaces of IoT devices need to be focused on in order to analyze the existence of possible threats and vulnerabilities, among which the general IoT attack categories are depicted in Figure 1, and major attack targets are depicted in Figure 2.

Based on the current literature related to security violations among IoT devices, the aforementioned attacks in Figure 2 play a major role in weakening security and making the complete system unavailable to intended users. Distributed denial-of-service (DDoS), malware, and Man-in-The-Middle (MiTM) attacks focus on violating confidentiality and availability constraints and making IoT devices and communication channels vulnerable.

In addition to Man-in-the-Middle (MiTM) and distributed denial-of-service (DDoS) attacks, there are other types of attacks that can disrupt the normal functioning of IoT devices and their applications. These attacks include denial-of-service, jamming, Sybil, black hole, worm hole, and malware attacks. Among these attacks, DDoS attacks are considered the most vulnerable as they disrupt and corrupt the complete functioning of the entire system, causing more damage and taking more time to recover compared to the severity of other attack categories.

In this paper, we have focused on DDoS and false data sharing attacks as the major attack categories and proposed a comprehensive solution to mitigate these attacks by using various methods such as simple network management protocol (SNMP), Kullback–Leibler distance (KLD), access control rules (ACL), and moving target defense (MTD) techniques. The SNMP is used to closely monitor the abnormal patterns of incoming network traffic based on changes in the values of SNMP management information base (MIB) variables. DDoS detection is carried out by analyzing the incoming attack patterns and packet structure of attack requests and formulating ACL rules to block known DDoS patterns. The MTD techniques are applied to harden the attack target and reduce the attack probability. The proposed method has been validated by estimating attack probabilities and switching delays in an experimental testbed simulation on the Amazon Web Service (AWS) platform. Results have shown that the proposed method is effective in reducing the attack probability to a minimum of 0.2%.

Furthermore, the proposed solution is designed to be flexible and adaptable to different types of IoT environments and can be easily integrated with existing systems. It also provides a comprehensive and effective approach to detecting and mitigating DDoS and false data-sharing attacks in IoT environments.

## 2. Technical Background

The key security concerns in the IoT environment [6] are classified into implementation, privacy, network infrastructure, security threats, malware, authentication, and authorization-related challenges. Violation of IoT-related security focus merely related to the areas of privacy and confidentiality among heterogeneous management and network capacity constraints. Possible security issues include securing IoT architecture, active detection, and protection of DoS and DDoS, standards, methods, or tools for managing all user identity and objects. A few issues related to the private domain are personal information control, improvement of privacy technologies and related rules, protection of exchanged sensitive information over the communication medium, and confidentiality of stored messages. The widespread adoption of the cloud to effectively carry out the collection, storage, and analysis of IoT data paves the way for more associated open challenges [7], which disrupt authorized access, retrieval, and extraction of information from the cloud.

The goal of this paper [8] is to identify the security challenges and key issues that are likely to arise in the IoT environment in order to guide authentication techniques to achieve a secure IoT service. Denial-of-service (DoS) [9] is considered to be the most dominant and devastating in the IoT environment. The attackers could be using flooding attacks in order to exhaust system resources such as CPU, memory, and bandwidth. With the adoption of numerous techniques, the attacker’s target is to flood the network with bogus packets [10,11,12] and hence block legitimate or trusted users from utilizing the usual services.

Replay attack [13] targets the authentication and key exchange-related protocols in order to capture or store either a whole session or a fragment of a session in legitimate traffic. On gaining trust over the public network, the attacker sends the captured message in order to indicate participation in the original session. A replay attack is mentioned as a security weakness or vulnerability in the authorization procedure for accessing stored data. To handle a replay attack, the current scenario uses three types of solutions, including timestamp, nonce, or challenge-response mechanisms. The freshness of a message is identified and tracked by using the concept of timestamp, where the purpose of the nonce is the generation of random digits and comparing the same for granting access. Challenge-response measures attempt to test the pre-shared secret values between the user and the target system or entity.

The password-guessing attack [14,15] occurs by overhearing the communication channel by exploring weaknesses in numerous authentication protocols. This type of attack could take place either in online or offline mode. The attacker will be able to guess all possible passwords in order to succeed in the authentication process. The main aim of a spoofing attack is to make the servers trust that the attacker is one of the authorized entities.

**Figure 3:**
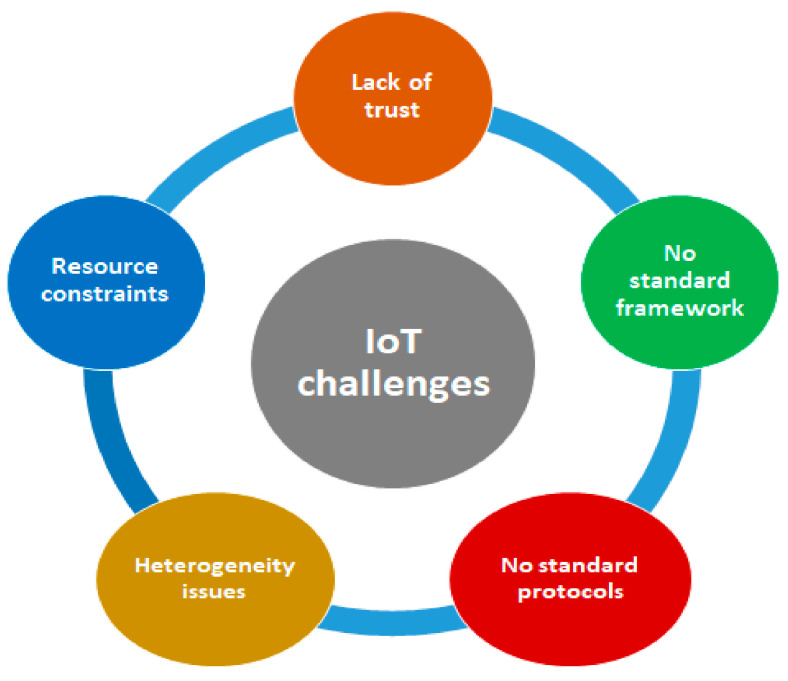
IoT challenges.

Various categories of distributed denial-of-service (DDoS) attacks, along with their impact on IoT devices, are discussed [16], along with detailed mitigation models. It deliberately discusses the classification of different intrusion detection systems, anomaly detection techniques, different intrusion detection models related to datasets, various machine learning and deep learning methods for pre-processing data, and malware detection is carried out. Most of the security challenges are specific to the issues related to IoT devices and are listed below in Figure 3.

There are many categories of DDoS attacks specific to IoT devices namely, TCP SYN flood, tear drop, Smurf, ping of death, and botnet attacks. The main classification of DDoS includes reflection and amplification attacks. The main difference between these two attack categories could be analyzed by observing the size of request and response packets. The size of the response is n times bigger than the request size in an amplification attack, whereas the size of the response is equal to the request size in reflection-based attacks. Attackers will be utilizing common vulnerabilities for launching DDoS attacks:

- Insecure connection;
- Weak password;
- Firmware updates;
- Software vulnerabilities;
- Data handling-related vulnerabilities.

Various anomaly detection techniques [17,18] used for detecting the presence of malware in IoT devices are signature-based detection and anomaly-based detection. The former detection pattern is not a successful one as the bots keep on changing their signature pattern, while the latter helps in tracking behavioral changes between the normal and botnet traffic. Other approaches related to community-based anomaly detection [19,20,21] focus on identifying bots based on the communication graph. A bad neighborhood is one of the methods utilized in phishing and spam detection to identify the cluster of IP addresses that performs malicious activities over a period of time. The various authentication techniques for securing IoT devices are detailed in Table 1.

## 3. Dimensions of Security Threats in IoT

The security threats and challenges associated with IoT are more prevailing as, according to a recent survey of IoT analytics, by 2025, there will be 30.9 billion [22] connected devices in the world. The increasing security vulnerabilities and cyber-attacks block many users from utilizing IoT devices. IoT-related security problems are more prevalent in healthcare and logistic-related domains [23]. The security challenges associated with IoT data while operating in a cloud environment could be generalized based on the analysis of common threats prevailing in the current scenario, which are listed below:

- Software vulnerabilities;
- Firmware vulnerabilities;
- Insecure communication channel;
- Data leaks from IoT systems;
- Malware risks;
- Cyber-attacks.

The possible causes for the occurrence of such threats in IoT-associated cloud environments are due to the following:

- Lack of computational capacity;
- Poor access control techniques;
- Limited budget to carry out testing;
- Limited budget to ensure firmware security;
- Lack of regular patches;
- Lack of periodic upgrades;
- Technical limitations of IoT devices;
- Unavailability of software updates for older IoT devices;
- Ineffective protection from physical attacks.

**Figure 4:**
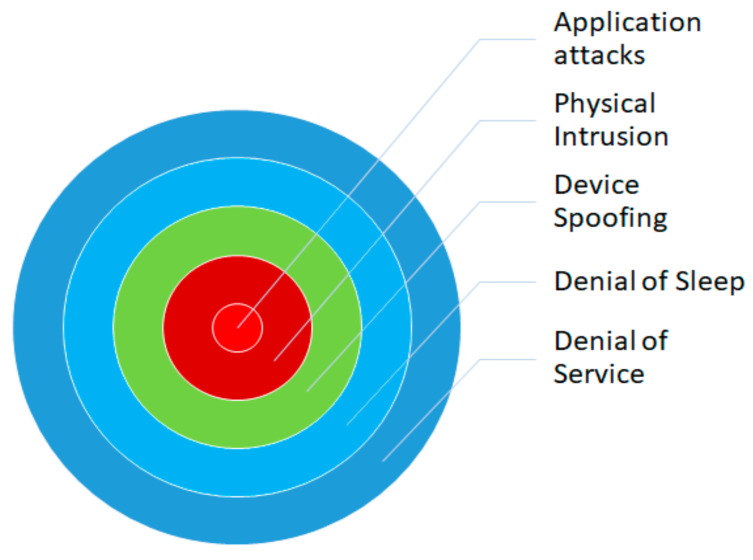
Cyber-attacks in IoT environment.

One of the most dangerous threats which happen due to an insecure communication medium is the Man-in-the-Middle (MiTM) attack. On installing malware or by changing the device’s functionality, MiTM is launched if the device does not use any encryption or authentication mechanisms. Man-in-the-Middle (MiTM) attacks are a significant threat to the security of IoT systems due to their reliance on insecure communication mediums. These attacks involve the attacker intercepting and altering communication between two parties without their knowledge or consent. This can be accomplished by installing malware on a device or altering its functionality. IoT systems are particularly vulnerable to MiTM attacks because they often lack robust encryption and authentication mechanisms. As a result, attackers can easily intercept and modify communication between devices, making it difficult for users to detect and prevent these attacks. IoT systems are prone to various cyber-attack categories, as depicted in Figure 4. Application attacks target vulnerabilities in the software or firmware of an IoT device. This can include SQL injection, cross-site scripting, and command injection attacks. Physical intrusion involves physically accessing an IoT device to extract information, install malware, or disrupt its operation. An attacker may use tools like lock picking or physical access to the IoT device to perform these attacks. 

Device spoofing involves tricking a device into connecting to a fake device or network, allowing the attacker to intercept or modify communication. This can be done through a technique known as “rogue access points” or “evil twin” attacks. Denial of sleep involves preventing IoT devices from entering a low-power state, which can cause them to consume more energy and potentially shorten their lifespan. Denial of service involves overwhelming a device or network with a flood of traffic, making it inaccessible to legitimate users. This can be done through a distributed denial-of-service (DDoS) attack, where multiple devices are used to flood the target.

These attacks are often interconnected, with one layer of security being compromised, providing an easy pathway for a DDoS attack to be launched.

**Figure 5:**
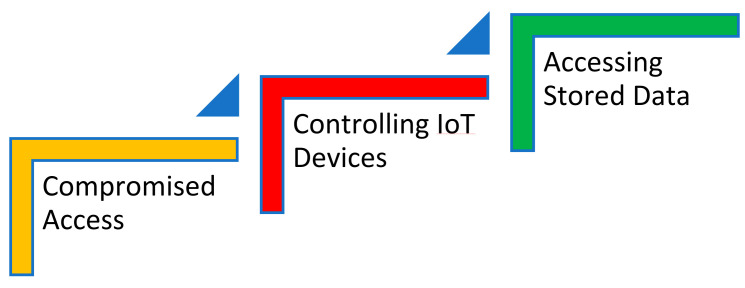
Stages of IoT threats in general.

On careful analysis and deep inspection of existing cyber-attacks, general attacks, and device-specific threat categories of IoT in a cloud environment, the below structure, as depicted in Figure 5, is formulated. Irrespective of the type of cyber-attack in the cloud-assisted IoT environment, the below steps remain the same.

**Figure 6:**
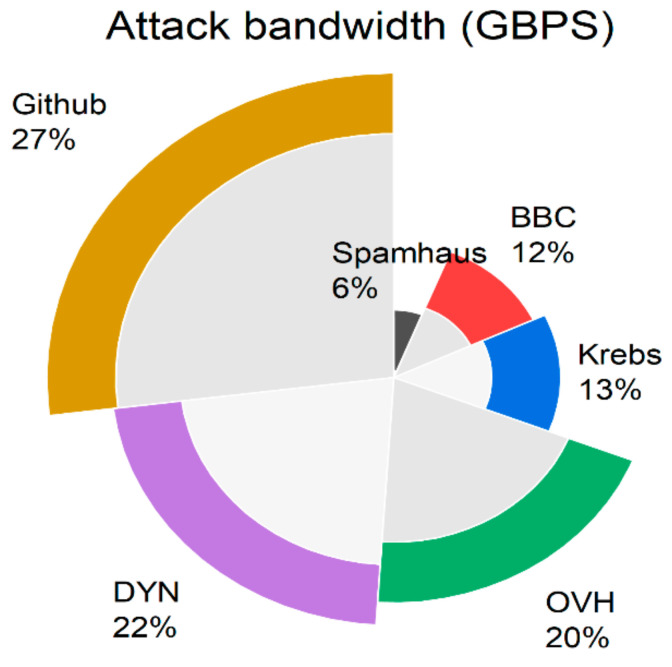
DDoS statistics in various IoT platforms.

**Figure 7:**
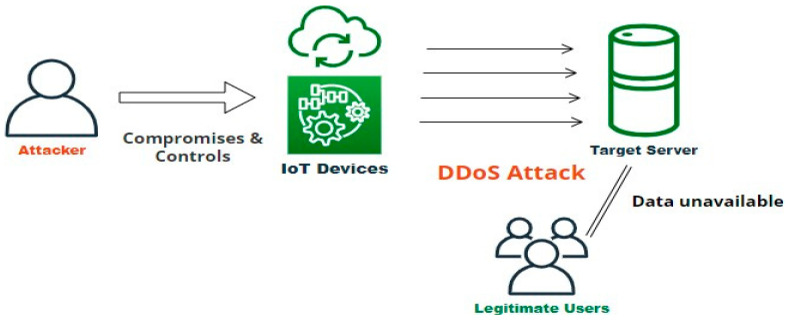
DDoS attack in IoT platform.

Among other attack categories in the IoT environment, DDoS seems to be more prevalent, which is evident from the below statistics depicted in Figure 6. DDoS is more devastating as it makes the complete IoT devices inaccessible and unavailable for the legitimate user community. The prevalence of DDoS attacks in IoT platforms and their underlying structure are depicted in Figure 6 and Figure 7. Figure 6 clearly illustrates the bandwidth affected in the IoT platform due to the launch of a DDoS attack. The ultimate reasons for DDoS attacks are the insecure communication medium and unsecured data in a cloud environment. The proposed works address both by formulating an efficient architecture.

Due to the prevalence of DDoS attacks in IoT environments, this paper suggests the application of moving target defense strategies to make the attack target harder and to decrease the attack probability by applying various MTD techniques, diversity, and migration.

## 4. IoT Attack Detection Procedure Using SNMP and Kullback–Leibler Distance (KLD)

**Figure 8:**
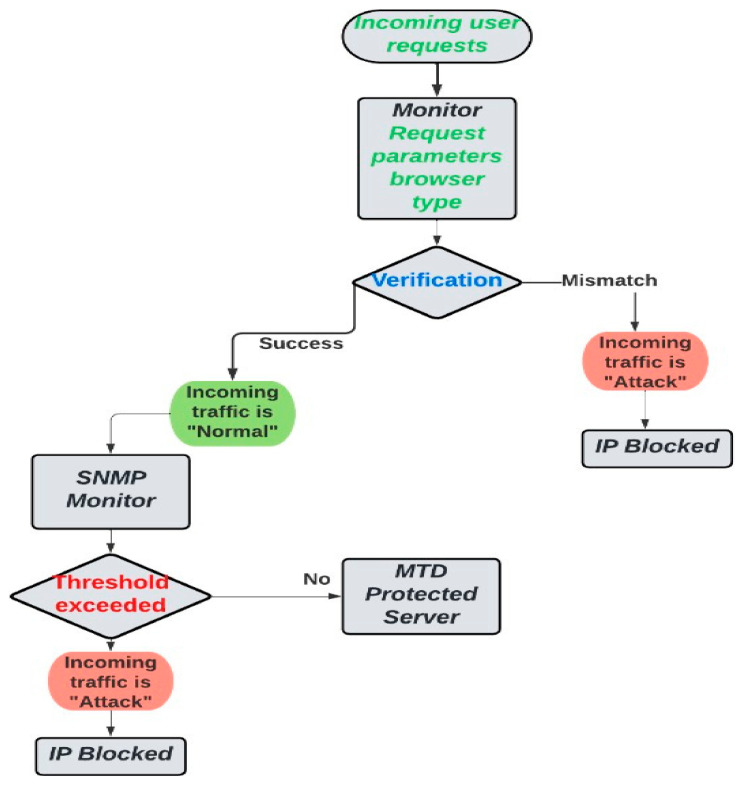
Overall DDoS detection using SNMP, mitigation using ACL.

The most devastating threat to IoT devices, namely DDoS and false data injection, is chosen to provide mitigation solutions in terms of access control list and moving target defense. Detection of DDoS targeting IoT devices is identified with the help of a simple network management protocol (SNMP) [24,25]. SNMP is an application layer protocol that is merely adopted to track the normal functioning of network devices. In the DDoS pattern analysis experiment, SNMP features are adopted by deploying SNMP agent and SNMP manager source codes in the experimental setup discussed in [26]. Based on the changes in SNMP MIBs tcpActiveOpens, tcpPassiveOpens, and tcpAttemptFails, the incoming traffic is categorized as “normal” or “attack”, which is depicted in Figure 8. Based on the observations from SNMP and further analysis of incoming request headers [27], ACL rules are formulated, which work by blocking unknown, malformed headers and unknown reference links. On detecting DDoS attacks using SNMP MIBs, access control rules are formulated as a basic Level 1 prevention measure.

The detection of false data injection is done by estimating the Kullback–Leibler distance (KLD) between the original data (at the time of sending) and received data (after sending across the communication channel).

This method is proven to be most effective in the detection of abnormal traffic based on either the packet size or distribution statistics [28,29,30,31]. Probability distributions for data before sending in the network and after sending in the communication channel are carried out, and differences between them are analyzed using Equation (1). Information distance is represented as the divergence between two probability distributions. The information divergence between the probability distributions X and A where $X=X_{1},X_{2},\ldots ,X_{n}$; $A=A_{1},A_{2},\ldots ,A_{n}$ of order α could be calculated as:

$$D_{\alpha }\left(X∥A\right)=\frac{1}{\alpha -1}\log_{2}\left(\sum_{i=1}^{n}p_{i}^{\alpha }q_{i}^{1-\alpha }\right),\;\alpha >0$$

Detection of false data injection attacks on the data collected from IoT sensors could be identified with the help of Kullback–Leibler distance. Larger KLD reflects the variation in probability distributions of the measurements from historical data. It is a natural distance function from a true probability distribution X to a target probability distribution A. Based on the deviation in final equation values, it could be verified if any false data is added along with the original one or not.

## 5. IoT Attack Mitigation Procedure Using ACL and MTD in AWS

The complete setup is configured in the Amazon Web Service (AWS) console to represent various proxy and web server configurations. For mitigation against DDoS, two levels of control measures are enforced by adopting the access control list in Level 1 and moving target defense-based migration concepts in Level 2. All the initial parameters specific to legitimate user requests are analyzed in detail, based on which the specific functionalities are identified in pre-defined ACL rules.

**Figure 9:**
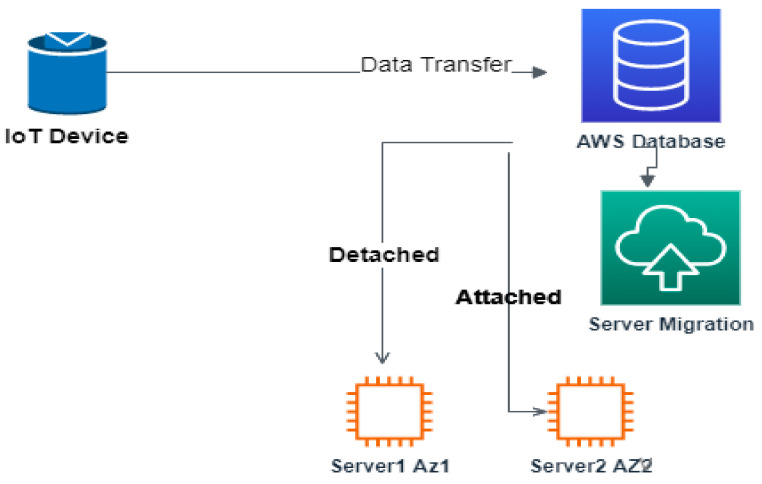
Securing IoT data in the cloud using MTD.

The main purpose of adding ACL rules in Level 1 is to filter DDoS attack traffic related to user-agent, header filed, request size, request count, and IP address. The requests which get validated and proven to be legitimate in Level 1 will be moved to Level 2. To secure the main or target server from crashing due to unwanted malicious incoming traffic, server hardening is carried out, which in turn decreases the DDoS attack probability by hiding the IP of the main server by maintaining servers in different availability zones and with the migration process. The target servers are made dynamic by applying the concept of MTD diversity. The location of the target server changes from time to time across various availability zones in order to withstand attacks by applying the concept of migration, which is depicted in Figure 9.

The AWS server instance plays a crucial role in maintaining the data that is transferred from IoT devices. In order to ensure the confidentiality of this data, the concept of server migration is applied. This is done to prevent an attacker from compromising the data by gaining knowledge of the IP address and other protection mechanisms of the server using port scanning tools.

**Figure 10:**
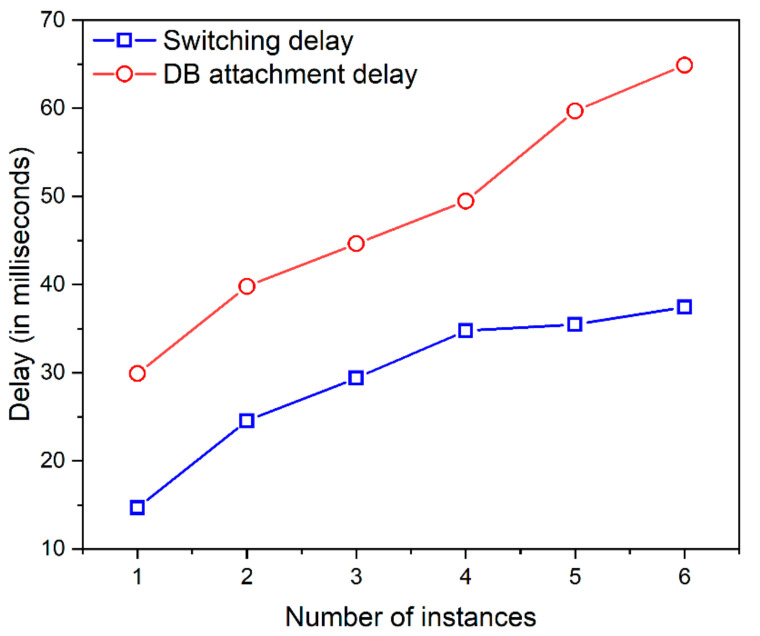
MTD delay estimation metrics observed in AWS.

**Figure 11:**
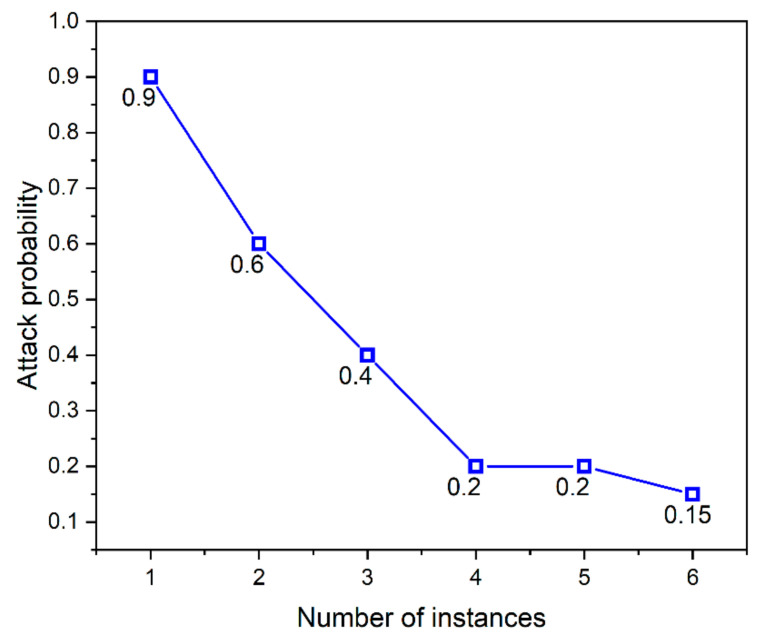
Attack probability on applying.

The moving target defense (MTD) technique is applied to achieve more security by migrating the server instance from time to time. The migration process does not cause any delay or connection issues whenever there is a legitimate attempt because the AWS server instance in different availability zones alone remains attached or detached from the database instance. This is validated by the graph depicted in Figure 10 and Figure 11.

In Figure 10, the *x*-axis represents the number of instances chosen in AWS, and the *y*-axis represents the switching delay in milliseconds. The switching delay indicates the requests directed from one instance to another. The y-axis also represents the DB attachment delay in milliseconds which indicates the time taken to attach DB from one instance to another.

In Figure 11, the *x*-axis indicates the number of instances in AWS, and the *y*-axis indicates the attack probability reduction with an increase in the number of instances. It is observed that on increasing the number of AWS instances and applying the concepts of MTD, the attack probability is reduced to a minimum of 0.15% as the probability of occurrence of an attack. The chosen MTD method outperforms the existing schemes [22,31] in reducing the attack probability from 0.5% to 0.15%, the switching delay reduced to 0.076 s from 1.2 s, and the DB attachment delay also decreased on comparing the existing literature from 1.4 s to 0.032 s.

Therefore, the methods applied in the proposed method are effective in mitigating the IoT-based DDoS and false data-sharing attacks with a considerable increase in performance metrics. The result shows that the MTD technique is effective in reducing the attack probability and improves the performance of the system by reducing the delay of switching and DB attachment.

## 6. Conclusions

IoT technology plays a vital role in today’s digital, interconnected scenario. Ensuring the confidentiality and security of data from IoT devices is crucial for the success of the entire IoT model. In this paper, we discussed the detection and mitigation of DDoS and false data injection attacks in IoT environments. The detection of these attacks was carried out using simple network management protocol (SNMP) and kernel learning detection (KLD), whereas the mitigation was done using access control lists (ACL) and moving target defense (MTD) techniques. The proposed techniques were found to be more accurate and efficient than existing security techniques.

The focus of future work should be extended to the proposed techniques to detect and mitigate other types of IoT attacks. The communication channel is secured by maintaining ACL lists and SNMP monitors, while the stored data in AWS instances is maintained using MTD techniques such as diversity and migration. Both these techniques help in maintaining the dynamic IP address of the server and provide an added layer of security by not giving any clue about the location of the data. The processing delay due to migration is negligible, as evident from the experimental results discussed in the paper.

In conclusion, the proposed techniques provide an efficient and effective solution for detecting and mitigating DDoS and false data injection attacks in IoT environments. The proposed techniques are not only accurate but also improve the performance of the system by reducing the delay of switching and DB attachment. As IoT technology continues to evolve and expand, it is essential to develop robust security mechanisms to protect against various types of attacks and ensure the confidentiality of data.

## Figures and Tables

**Table 1:** Summary of Authentication Techniques for securing IoT devices.

Sl. No	Method	Benefit	Limitations
1	One Time Password	Secures communication in an IoT environment based on mechanisms of time synchronization, hash functions, cryptography, and RSA.	Vulnerable against full key-recovery attacks on HMAC/NMAC-MD4 and NMAC-MD5.
2	ECC-based mutual authentication	It is more efficient for systems that pose limited memory and processing capabilities.	Hyper-elliptic curve cryptography technique could be adopted for more security.
3	ID and password-based authentication	Client-server authentication architecture is strong.	Storage of user data in the server and method of protecting the same from insider attacks is the known vulnerability. If users forget their authentication parameters, they need ways to retrieve the same. It is not suitable to save personal ID in an electronic device even if it is not connected to any network. Transmission of user ID in the public network is another challenge to be focused on.
4	Certificate-based authentication	It is commonly used to verify a user’s identity in banking applications. It provides more security and device certificate processing.	It is not suitable for IoT objects as it necessitates a high processing resource that is not always in IoT devices.
5	Block chain	Highly sustainable. Authentication protocol is secure. Verification ensures a high level of confidence in recorded data. Multiple and different authentication methods are used. Various cryptography types are used. Random numbers are used to ensure the freshness of the message.	It is considered effective only if it is lightweight and fulfills security requirements. Running time and processing time need to be considered due to the limitations of IoT devices.

## Data Availability

Not applicable.
